# Deubiquitinating Enzymes in Coronaviruses and Possible Therapeutic Opportunities for COVID-19

**DOI:** 10.3390/ijms21103492

**Published:** 2020-05-15

**Authors:** Valentino Clemente, Padraig D’Arcy, Martina Bazzaro

**Affiliations:** 1 Department of Experimental, Diagnostic and Specialty Medicine, University of Bologna, 40138 Bologna, Italy; 2 Department of Biomedical and Clinical Sciences (BKV), Linköping University, SE-58183 Linköping, Sweden; padraig.darcy@liu.se; 3 Masonic Cancer Center and Department of Obstetrics, Gynecology and Women’s Heath, University of Minnesota, Minneapolis, MN 55455, USA; mbazzaro@umn.edu

**Keywords:** DUBs, coronavirus, SARS, SARS-CoV2, papain-like protease, PLP inhibitors, COVID-19, COVID-19 therapy

## Abstract

Following the outbreak of novel severe acute respiratory syndrome (SARS)-coronavirus (CoV)2, the majority of nations are struggling with countermeasures to fight infection, prevent spread and improve patient survival. Considering that the pandemic is a recent event, no large clinical trials have been possible and since coronavirus specific drug are not yet available, there is no strong consensus on how to treat the coronavirus disease 2019 (COVID-19) associated viral pneumonia. Coronaviruses code for an important multifunctional enzyme named papain-like protease (PLP), that has many roles in pathogenesis. First, PLP is one of the two viral cysteine proteases, along with 3-chymotripsin-like protease, that is responsible for the production of the replicase proteins required for viral replication. Second, its intrinsic deubiquitinating and deISGylating activities serve to antagonize the host’s immune response that would otherwise hinder infection. Both deubiquitinating and deISGylating functions involve the removal of the small regulatory polypeptides, ubiquitin and ISG15, respectively, from target proteins. Ubiquitin modifications can regulate the innate immune response by affecting regulatory proteins, either by altering their stability via the ubiquitin proteasome pathway or by directly regulating their activity. ISG15 is a ubiquitin-like modifier with pleiotropic effects, typically expressed during the host cell immune response. PLP inhibitors have been evaluated during past coronavirus epidemics, and have showed promising results as an antiviral therapy in vitro. In this review, we recapitulate the roles of PLPs in coronavirus infections, report a list of PLP inhibitors and suggest possible therapeutic strategies for COVID-19 treatment, using both clinical and preclinical drugs.

## 1. Introduction

A pneumonia of unknown cause was detected in Wuhan, People’s Republic of China and first reported to the World Health Organization (WHO) Country Office on 31 December 2019. The pathogen was subsequently discovered to be a novel coronavirus (CoV) and named severe acute respiratory syndrome (SARS)-CoV2, based on sequence similarity with members of the β-coronavirus family, such as SARS-CoV and MERS-CoV, two highly pathogenic coronaviruses responsible for previous epidemics of Severe Acute Respiratory Syndrome and Middle-East Respiratory Syndrome, respectively [1,2,3,4]. Genomes of coronaviruses are typically organized with a certain degree of similarity, suggesting analogous pathogenic mechanisms. Other human coronaviruses include the NL63, 229E, OC43 and HKU1, which are usually responsible for common colds and/or other respiratory infections in children and older or immunocompromised individuals [5,6].

On 11 February 2020, the WHO named the new coronavirus disease: COVID-19, alias COrona-VIrus Disease 2019. Two months later, following a large spread outside China, with epidemic foci in South Korea and Japan and a massive outbreak in Italy, the WHO declared COVID-19 a pandemic [7]. Thus far, this is the first highly pathogenic coronavirus epidemic to reach these proportions.

Mortality data and severity assessments made available by the Chinese government and the WHO indicate that about 80% of affected individuals have symptoms similar to those of mild seasonal influenza. The remaining 20%, however, develop viral interstitial pneumonia, requiring hospitalization. In approximately 5% of these patients, the pneumonia is critical and requires intensive care. Overall mortality ranges between 0.5% and 5%, with a clear positive correlation with age and other comorbidities [8]. 

Based on these numbers, the majority of the world’s health care systems are ill-equipped to deal with the surge of patients requiring hospitalization without taking additional measures to prevent system collapse. There is an immediate concern that the mortality rate could increase further due to a lack of available intensive care unit beds required to treat severe cases. Furthermore, since no large clinical trial has been possible considering the timescale of the pandemic, there is no consensus on how to treat severe cases of COVID-19 viral pneumonia. Thus far, all evidence of treatment options are derived from a few case experiences during the SARS and MERS epidemics, from administrations made in special cases based on analogy with other diseases, intuitions and experimental therapy of some pathological features/symptoms [4,9,10,11,12]. As we are in the rising part of an epidemic time/contagion graph, with a significant proportion of the world’s population under restrictions or quarantine, the identification of therapeutic options for treating severe cases of COVID-19 is of paramount importance.

The ubiquitin proteasome system (UPS) is a key regulator of protein homeostasis. The pathway to proteasomal degradation consists of a group of enzymes called ubiquitin (Ub) ligases, that attach ubiquitin moieties to target proteins. The addition of ubiquitin serves as a highly specific destruction tag, where ubiquitinated proteins are trafficked to the proteasome for degradation. A reverse pathway involving ubiquitin removal also exists and is mediated by the action of deubiquitinating enzymes (DUBs) that catalyze the removal of Ub from tagged proteins [13]. While proteasome targeting tends to involve poly-Ub chains containing Ub moieties linked via isopeptide bonds at lysine residue 48 (K48) of Ub, other Ub linkages (particularly K63) and Ub-like modifications (i.e., sumoylation, NEDDylation and ISGylation) are involved in signal transduction and the regulation of immune responses. It is of no surprise that viruses, including coronaviruses, often use modulation of ubiquitin and ubiquitin-like modifiers to evade the host cell immune response [14,15]. 

Each *Coronaviridae* family member codes for DUBs, named viral papain-like proteases (PLPs), which remove ubiquitin from target proteins and alter cellular pathways important for infection. Some members encode two, but, SARS, MERS CoVs and the novel SARS-CoV2 [3] only encode one, named SARS-CoV PLP, MERS-CoV PLP and SARS-CoV2 PLP respectively. For many coronaviruses, viral PLPs have been studied extensively and shown to play a crucial role during viral infection of the host cell. These enzymes are multifunctional and in addition to their DUB activity, also containing intrinsic cysteine protease and deISGylating activity that are required for viral replication and the evasion of host responses [5,6]. 

The deISGylating activity of PLPs is similar to DUB activity and involves deconjugating interferon (IFN)-stimulated gene (ISG)-15 moieties from tagged proteins. ISG15 is a small Ub-like peptide that can be covalently attached to target proteins in a mechanism similar to Ub, resulting in a large number of regulatory effects. ISG15 is largely stimulated during antiviral responses, and although its broad functions are not fully elucidated, it acts as an effector and a regulator of the host cells innate immune response during viral infections [16,17].

Since viral PLPs are used by coronaviruses to both replicate and to antagonize the innate immune response, they are considered important therapeutic targets for coronavirus infections and thus may be of interest for future COVID-19 treatment strategies. In this review, we report an up-to-date description of coronaviral PLPs functions and their inhibitors, and provide possible therapeutic strategies for COVID-19 treatment, using both clinically approved and preclinical drugs.

## 2. Methods

The following keywords: “DUBs in coronavirus” “DUBs in SARS-CoV” “SARS-CoV PLP role” “PLP activity” “PLP inhibitors” “PLP in SARS-CoV2” and “SARS therapy”, were used in a literature search of the PubMed database. The cut off dates were 2005 for the pathogenesis dissertation and 2013 for novel drugs. 

## 3. Results

### 3.1. Role of PLPs in Coronaviruses Replication and Infection

Viral PLPs are highly conserved among the *Nidovirales* order members [5] and the structure of some relevant coronaviruses PLPs has been elucidated using crystallography and the enzymatic assays [18,19,20,21,22,23,24,25]. The multifunctional activities of PLPs, namely as cysteine proteases, DUBs and deISGylating enzymes, play two important roles in coronavirus pathogenesis: the first involves the production of non-structural proteins (nsp) required for the replication process and the second consists of blocking the innate immune system of the infected host cell.

#### 3.1.1. PLPs as Cysteine Proteases

**Figure 1:**
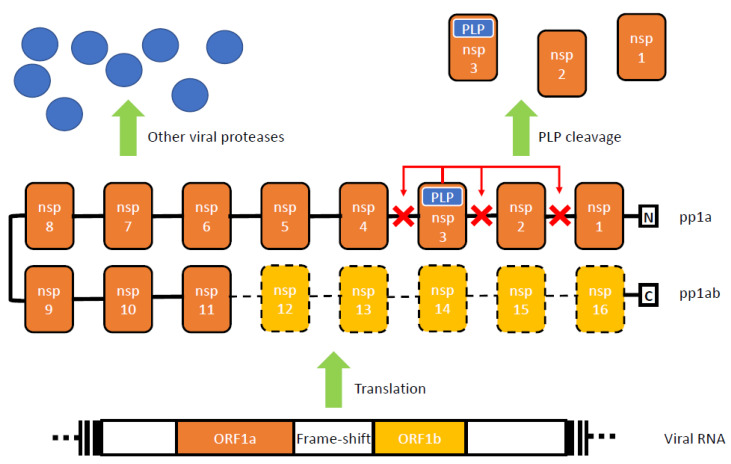
Schematic representation of the role of severe acute respiratory syndrome (SARS)-coronavirus (CoV) papain-like protease (PLP) role during infection. The figure represents the early roles of SARS-CoV PLP during the replication phase. The replicase/transcription complex (RTC), is coded by two open reading frames, ORF1a and ORF1b, that, with a ribosomal frame-shift mechanism, allow for the translation of two polyproteins (pps): pp1a and pp1ab. Pps are in turn constituted by 16 non-structural proteins (nsp), 1-11 for pp1a and 1-16 for pp1ab. SARS-Cov PLP is encoded within nsp3 and is responsible for the cleavage of the N-terminal portion of pps, cutting the bonds between nsp1/2, nsp2/3 and nsp3/4.

PLPs play their first role during the early replicative phase of coronavirus infection. After the virus enters the host cell, a replication/transcription complex (RTC) is required to orchestrate the replication of the viral units in the cytoplasm. Here, the PLPs’ cysteine protease activity is essential for the cleavage of the N-terminal segment of the RTC polyprotein (pp). Specifically, the RTC is coded by two open reading frames (1a and 1b), that, with a ribosomal frame shift mechanism, lead to the transcription of two polyproteins: pp1a, that features the nsps from 1 to 11, and the larger pp1ab, that, in addition, contains nsps 12 to 16. The pps need to be processed correctly into the nsps, which are the active elements of the RTC. PLPs are encoded within nsp3 and free the N-terminal nsps of the RTC, while the remaining units are processed by the three chymotrypsin-like cysteine protease (3CLP alias main protease), which may also serve as an attractive therapeutic target [5,6,26]. SARS and MERS CoVs are known to adhere to this model and a visual description of the SARS-CoV PLP role as a cysteine protease is given in Figure 1.

#### 3.1.2. PLPs as DUBs

The host cell innate immune response to viruses begins once the viral nucleic acids are sensed by pattern recognition receptors such as Toll-like receptors (TLR) and retinoid inducible gene I (RIG-I). This event triggers signal cascades, including kinases such as TANK-binding kinase-1 (TBK1) and inhibitor-kB kinases (IKKs), leading to activation of transcription factors such as interferon-regulatory factor-3 (IRF3) and the nuclear factor kappa-light-chain-enhancer of activated B cells (NF-kB). These proteins translocate into the nucleus and serve as transcription factors by binding to promoter elements in target genes, leading to the expression of several cytokines, including type I IFNs (as α and β IFNs), which in turn will trigger a huge number of intra and extra cellular processes leading to activation of the innate immune response [27].

**Figure 2:**
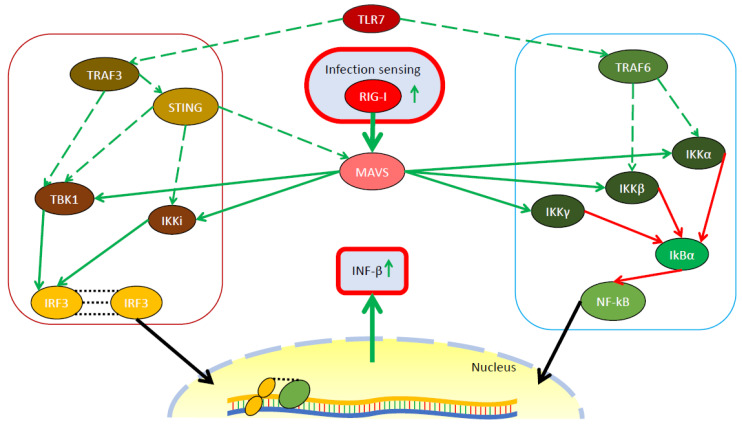
Schematic representation of the effect of SARS-CoV PLP on host cells’ immune system. The two proposed immune pathways affected by the SARS-CoV PLP are schematically represented here. Once the virus is detected by pathogen recognition receptors as RIG-I, the signal is transduced via MAVS to the activating kinases of the transcription factors: IRF3 and NF-kB. TBK1 and IKKi phosphorylate IRF3 and thus trigger its dimerization and nuclear translocation. IKKα-γ free NF-kB, that moves to the nucleus, by phosphorylating its inhibitor, IkBα. Activated STING and TRAF3 form complexes with IRF3 upstream regulators and thus increase the activation state. Finally, IRF3 and NF-kB promote the activation of the type I INF antiviral response. The PLP DUB activity antagonizes these pathways at multiple steps, resulting in a global antagonism of the signaling and lower activation of INF-β. For a detailed description of the interplays between the PLP and the proteins, the reader should refer to the text.

Coronaviruses infections, such as SARS, generally include a dysregulation of the immune system in their pathogenic features at systemic level [28,29]. Indeed, early research has shown that active SARS-CoV replication does not stimulate INFs production in cell culture [30]. Since it is known that cellular antiviral pathways include (de)ubiquitination within their regulatory mechanisms [14,15], PLPs are believed to contribute to infection pathogenesis by using their intrinsic DUB and deISGylating activities to antagonize the activation of the host cell innate immune response [5]. Specifically, PLPs use their DUB activity to interfere with the proteins that mediate the intracellular sensing and signaling of viral infection, therefore leading to a dysregulation of the immune pathways, such as the IRF3 and NF-kB pathways, that in turn, results in a decrease in the antiviral response. The DUB activity of PLPs is usually broad spectrum and not selective for specific ubiquitin linkage types, such as K48 or K63 [31,32,33]. Since DUB activity was first hypothesized as an important component of SARS-CoV PLP in 2005 [34], a number of studies investigating the DUB activity of coronaviruses PLPs and their effects on host cell innate immunity have been performed [35,36,37,38]. A map of the pathways that will be discussed in the next paragraph is given in Figure 2.

While the MERS-CoV PLP has been reported to have analogous effects [31,39], the SARS-CoV PLP DUB activity is the best characterized and has been demonstrated to antagonize the IRF3 pathway at many steps. This PLP was first reported to inhibit the RIG-I and TLR3 induction of the IFN-β [32,40], to block the phosphorylation and dimerization of IRF3 by interacting with its upstream regulators [40,41] and to directly deubiquitinate IRF3and thus disrupt its IFN-β promoter activity [42]. However, recent studies, highlight a K63 Ub DUB dependent broad interaction with various, if not all, components of the IRF3 pathway, such as STING, TRAF3/6, MAVS and TBK1 [43,44,45]. Another important pathway influenced by the SARS-CoV PLP is NF-kB. Here, the DUB activity is known to be exerted upon IkBα, an inhibitor of NF-kB [41], and on the TNFα activation of NF-kB [21,45], also resulting in INF-β antagonism.

Considering that this PLP has more than one site to bind Ub [6] and a study showing that inhibition of PLP protease activity results in reduced NF-kB (but not IRF3) antagonism [32], it could suggest that IRF3 antagonism depends on DUB activity towards K63 linked Ub chains and NF-kB antagonism instead on DUB activity against K48 linked Ub chains [21,45]; a type of linkage that in vitro seems to be preferred by this PLP [18]. In summary, we suggest that PLPs antagonism of the antiviral response is generalized upon the pathways, rather than focused on their single elements, and counteracts various Ub modifications. 

Although additional studies may be required to fully elucidate the exact interactions through which each coronaviral PLP promotes viral infections, there are supporting evidences that the DUB activity of PLPs results in a reduction of the IFN mediated anti-viral response and in a broad spectrum downregulation of proinflammatory cytokines, i.e., CCL5 or CXCL10 [39].

#### 3.1.3. PLPs as deISGylating Enzymes

Once translated, type I IFNs activate the JAK-STAT pathway through the IFNs receptors via an autocrine and paracrine mechanism. This subsequently leads to the expression of a plethora of ISGs, either effectors, regulators or both, that embody the antiviral response [46]. ISG15 is a small Ub-like peptide that can be covalently attached to proteins by a three enzymes process, similar to Ub conjugation. During viral infections, ISGylation seems to target a high number of proteins at the translational level, either as an activating or inhibitory signal, while free ISG15 can also act as a cytokine [16]. The large number of potential targets in multiple signaling cascades makes its role difficult to delineate, however it is clear that it acts as an effector and a modulator of the antiviral response [17]. ISGylation can antagonize the formation of viral molecular complexes required for replication, activate molecules involved in immune pathways and act as a negative feedback for upstream factors. Interestingly, ISG15 deficiency in mice leads to increased and often deadly viral infections, but in humans seems to involve increased susceptibility to *mycobacteria* and dysregulated immune responses rather than viral infections [16].

There are well established evidence that coronaviral PLPs are able to deconjugate ISG15 moieties from proteins [19,21,24,25,39] and that this enzymatic activity is conserved among the species [5], but, since the detailed mechanisms through which ISG15 exerts its antiviral activity are still not completely elucidated, the exact consequences of PLPs deISGylating activity in coronavirus infections are still poorly understood. However, two mouse models demonstrate in vivo that ISG15 has antiviral properties against a murine hepatitis coronavirus [47] and that PLPs may disrupt them [48]. This does suggest that the deISGylating activity of PLPs is an important mechanism used by coronaviruses to counteract the host’s antiviral response.

Recent reviews highlight some reciprocal interplays between the IRF3 and NF-kB pathways and the ISGs [46,49], while previous studies have also suggested an activating effect of ISG15 on IRF3 [50]. We would thus propose that the global effects of PLPs on innate immune responses could result from a crosstalk and additive action of both their DUB and deISGylating activities.

#### 3.1.4. Other Effects of the SARS-CoV PLP

In addition to the above-described roles of PLPs enzymatic activities, other authors have demonstrated further effects on the infection pathogenesis. The SARS-CoV PLP has been shown to complex with SUD (SARS-unique domain) and stabilize the p53 E3 ligase RCHY1, leading to subsequent ubiquitination and the degradation of p53, thus resulting in a decrease of p53 mediated antiviral activity, similar to other human CoVs PLPs. [51]. Another report demonstrates a downregulation of ERK1 by the proteasome inhibitor MG132, as a way to suppress the INF-α stimulation by this PLP [52]. 

Last, but not least, this PLP was shown to induce an upregulation of the transcription and translation of transforming-growth-factor (TGF)-β1 through ERK, which was inhibited by treatment with MG132. Noticeably, the treatment with the ERK1/2 inhibitor U0126 inhibited the activation of TGF-β1 activated genes, with an 8.4-fold reduction observed for type I collagen [53]. Moreover, two recent papers have further investigated these connections. The results of these studies contributed to the understanding of the molecular mechanisms underlying this feature of SARS-CoV PLP, confirmed the effects seen on human samples, and showed pro-fibrotic features induced in lung histology by this PLP in a mouse model. Taken together, these considerations suggest the connection of PLPs with collagen expression via TGF-β1 signaling and a pathogenic role of SARS-CoV PLP in inducing lung fibrosis [54,55]. Furthermore, in a previous study, MG132 was also shown to improve the lung histology in a SARS murine model [56] and removing the DUB activity of PLP2 in a murine hepatitis virus also resulted in a better liver histology if compared to controls [57], adding strength to this hypothesis (see below). 

#### 3.1.5. PLPs as a Molecular Target for Antiviral Therapy

Although many additional structural and non-structural coronavirus proteins may contribute to suppress the cellular responses to viral infections [58,59,60], the role of PLPs in the production of the replicase proteins and their location at the center of numerous signaling nodes suggest validity in targeting them as an anti-viral strategy. Collaterally, considering these effects of PLPs and other viral proteins on dysregulating the innate immune response, one may hypothesize that they could be at the origins of a mechanism that underlies the potential development of a cytokine storm [28,29,61].

While the activities of PLPs are usually conserved among the *Nidovirales* order members [5], to which coronaviruses belong, an enhanced PLP antagonism of innate immunity seems to be a peculiar characteristic of zoonotic coronaviruses such as SARS-CoV [62] and to be conserved among the highly pathogenic β-coronaviruses [39]. Since the PLP present in novel SARS-CoV2 shows a high degree of similarity to the better-known SARS-CoV [2,3,4], we speculate they might have similar roles in pathogenesis. Crystallographic analysis might clarify the mechanisms underlying the COVID-19 pathogenesis and provide a therapeutic target for the identification of inhibitors through high throughput molecular screening.

Based on this, we propose that PLP inhibitors should be evaluated as a possible therapeutic option for the treatment of COVID-19.

### 3.2. PLPs Inhibition in In Vitro and In Vivo Biological Models 

#### 3.2.1. In Vitro

Several in vitro studies support the idea that PLP inhibitors may be effective in reducing coronaviruses infection. For instance, a recent work evaluated the effects on SARS-CoV by substituting its native PLP with the one derived from a bat SARS-related CoV. Interestingly, the obtained SARS-CoV replicated 10.3-fold less than the wild type in human airway epithelial cells, but showed no differences in non IFN competent cells [62]. Meanwhile, several past independent studies showed that small molecule PLP inhibitors can inhibit SARS-CoV replication in Vero E6 cells without toxic effects on the host [63,64,65,66]. 

Remarkably, in a 2017 work, its authors developed an innovative therapeutic strategy, using Ub variants, to inhibit MERS-CoV PLP in Vero cells. These protein-based inhibitors were shown to be highly selective for this PLP and to disrupt all of its enzymatic functions (pp processing, DUB and deISGylating activity), leading to a four orders of magnitude decrease in viral titer [67]. As the authors themselves recognize, the only questionable feature in this therapeutic strategy was the requirement of sophisticated protein engineering techniques. However, such a suggestion should not be rejected, and may indicate that other bio-pharmacological techniques, such as monoclonal antibodies designed against SARS-CoV2 PLP or PROteolysis TArgeting Chimeras (PROTACs), capable of mediating the ubiquitin-proteasome mediated degradation of SARS-CoV2 PLP, may have merit.

#### 3.2.2. In Vivo

Despite promising results shown in mice with proteasomal inhibition in a SARS model [56], only two main studies on murine models specifically consider PLP inhibition in vivo. First, in a 2014 study, IFN-receptor knockout mice that lack ISGs, and are not able to survive Sindbis virus infections, were infected with chimeric Sindbis viruses, engineered to express ISG15 and either SARS-CoV/MERS-CoV PLPs or their inactive mutants. Mice infected with the mutated PLP virus showed a 76% survival, attributable to ISG15 restoration, while the wild type PLP virus infection resulted in more than 80% mortality [48]. The treatment, with a drug developed in the authors’ laboratory and displayed in another report [66], was specific for PLP and induced an increase in ISGylation, that led to an increase in survival to the wild type PLP virus. Unfortunately, there was no significative efficacy in the SARS-CoV mouse model [48]. An additional more recent work reported similar results. Here, a USP18 (a cellular DUB with physiological deISGylating activity) knockout mouse model, displaying increased level of ISGylation, was infected with a murine hepatitis coronavirus. Higher ISGylation levels were positively correlated with a delay in coronavirus replication, and, instead, negatively with the PLP2 levels in vivo. Although, PLP2 inhibition was tested in vitro, lower viral titers were observed [47].

The latest, and second main, PLP inhibition model in vivo is an engineered murine hepatitis coronavirus. The DUB, and not the protease activity, of a murine hepatitis coronavirus PLP2 was disrupted through structure guided mutagenesis. The obtained mutant coronavirus was tested in macrophages for its ability to elicit cells’ innate immunity and showed an increased activation of IFN. However, inoculation in mice only resulted in a mild attenuation of virulence, despite an improvement in histological features [57]. As an explanation, the authors hypothesized that the DUB activity of this PLP may not be necessary in vivo, or may only be required in some tissue-specific/environmental contexts. Remarkably, the authors reported difficulties in isolating a mutant virus without PLP DUB activity and claim this was due to the difficulty in separating it from other activities that resulted in no replication if disrupted. This difficulty, per se, may have more meaning than just a technical issue, and suggests that PLPs may be essential for viral replication.

In summary, these three mouse models may confirm in vivo the previously described individual roles of PLPs enzymatic activities in the pathogenesis of coronaviruses infections and give some promising insights on targeting PLPs as an antiviral strategy. However, thus far, the evidence of the therapeutic efficacy of targeting PLP in vivo remains questionable. The first study gave positive results in a SARS-adapted mouse model, but only a single compound was investigated, while the second did not consider the inhibition of PLP in vivo and gave insights on its pathogenic role. The third study was specifically aimed to investigate the role of the DUB activity of PLPs, rather than to evaluate the therapeutic efficacy of targeting PLPs. As far as we are aware, the number of in vivo studies on PLPs is limited. Therefore, in vivo, therapeutic-oriented, studies in more reliable models, such as those developed for SARS and MERS, are strongly recommended [68].

In vivo studies, thus far, do not support the evidence of antiviral efficacy in targeting PLPs. However, since we suggest in vivo studies, on the basis of the PLPs pivotal role in pathogenesis, of the positive results in vitro against coronaviruses replication and of some in vivo features, PLP inhibitors might deserve at least a further discussion. 

### 3.3. Therapeutic Opportunities for COVID-19 Using PLPs Inhibitors

As previously mentioned, a number of drugs are already used or under clinical evaluation for the treatment of COVID-19. This includes the anti-malaria agent hydroxychloroquine, several antiretrovirals, as well as interferon and tocilizumab [4,9,10,11,12]. Importantly, because the structure of the SARS-CoV and MERS-CoV PLPs has been solved using crystallography, this has allowed for high throughput molecular screenings for the identification of a number of PLP inhibitors, that showed activity against viral replication in cells [63,64,65,66]. Many active compounds against SARS-CoV PLP have been previously reviewed [69]. In a recent report, molecular modeling and high-throughput screening methods of 30,000 molecules were used to identify inhibitors against the MERS-CoV PLP. The research conducted to compound 6 and ZT626, which were found to be competitive inhibitors in silico [70]. 

Importantly, sequence analysis has shown a high degree of similarity between SARS-CoV PLP and SARS-CoV2 PLP [3,4]. This has prompted computational screening, which has led to a number of predicted SARS-CoV2 PLP inhibitors, both clinical drugs and natural derivatives, with pre-clinical and clinical potential [3]. Here we report a series of PLP inhibitors evaluated for other coronaviruses, that could be considered for the novel SARS-CoV2. A brief overview is given in Table 1.

#### 3.3.1. Plant Compounds Derivatives

Several additional natural compounds derivates have been demonstrated to have activity against PLPs. Six compounds were isolated from *Tribulus terrestris* fruits and acted as mixed type inhibitors with IC_50_ values of 15.6–70.1 µM against the SARS-CoV PLP protease activity [71]. The evaluation of derivatives from *Angelica keiskei* leaves led to the isolation of 9 chalcones and 4 coumarins, with inhibitory activity against both proteases of the same coronavirus. Of these, compound 6 (xanthoangelol E) has an IC_50_ of 1.2 µM against the PLP and 11.4 µM against 3CLP (a chymotrypsin-like protease, also proposed as a molecular target against coronaviruses). *Angelica keiskei* derivatives were also identified as inhibitors of PLP DUB and deISGylating activity [72]. Park et al., who proposed many natural derivatives against coronaviruses, recently reported similar results from *Broussonetia Papyrifera* root derivatives, including the MERS-cov proteases [73]. Lastly, the seeds of *Psoralea Corylifolia* have been another source of lead compounds, such as isobavachalcone and psoralidin, with activity towards the SARS-cov PLP [74]. Unfortunately, all of these molecules have thus far only been evaluated biochemically in vitro and not at more advanced pre-clinical development stages, but, encouragingly, the extracts of *Strobilanthes cusia* leaves demonstrated PLP2 inhibitory properties and positive antiviral results against the human coronavirus NL63 in cells [75]. Therefore, we suggest that further research should be performed.

In addition, a recent meta-analysis demonstrates a protective effect of flavonoids against upper respiratory tract infections [80] and resveratrol has been found effective against MERS-CoV infection in Vero E6 cells [81]. This could indicate that the use of plants extracts such as CYSTUS052, a polyphenols rich medication already clinically tested in preventing influenza symptoms [82,83], could possibly be helpful in coronavirus infections, without having significant side effects. 

#### 3.3.2. Cystatin C

Cystatin-C, a small endogenous polypeptide, that physiologically acts as a cysteine protease inhibitor and is used as a biomarker of kidney [84] or brain [85] injury, has antiviral properties [86,87,88] and was found to be reduced in Vero E6 cells infected with a porcine epidemic diarrhea coronavirus [89]. Furthermore, it has been used at slightly supraphysiological levels in a previous study to reduce viral titers of two human coronaviruses in cell cultures, where the authors hypothesized a PLP as target, [79] and internalization of Cystatin C in cells has been demonstrated [90]. On the basis of these considerations, we would propose further studies on cystatin C antiviral properties against highly pathogenic coronaviruses. i.e., investigate the outcome of COVID-19 in patients with high levels of cystatin C.

#### 3.3.3. Clinical Drugs

Within the category of clinically available drugs, thiopurine analogs, used to treat diseases such as cancer or autoimmune diseases, may have been overlooked. They have previously been shown to display activity as SARS-CoV PLP inhibitors in vitro [76,91] and with similar activities, either as monotherapy or in association with mycophenolic acid, been shown against the MERS-CoV PLP [77]. Due to their well described mechanism of action, the authors proposed in vivo studies, that have not been performed thus far [76,77]. In addition, as the thiocarbonyl group has been judged to be the active element of these compounds, they may also serve as models to develop safer and more effective molecules [76]. Indeed, since such drugs have immunomodulatory actions and are known to depress the immune system, we would suggest a degree of caution in their use in infectious diseases.

However, no evidence indicating a higher risk of COVID-19 in patients under chronic treatment with immunosuppressive drugs has been reported, although several physicians have expressed concern [92,93]. We agree with the need of further investigation and propose a retrospective analysis to be performed as soon as possible on COVID-19 patients databases, to evaluate both the risk in individuals on immunosuppressive agents and, possibly, the effects of thiopurine analogs (or rather their prodrugs as azathioprine) on the outcome. Moreover, other similar therapeutic strategies have been proposed in vitro, i.e., alpha-interferon and cyclosporin [94].

Severe and advanced cases of COVID-19 pneumonias often involve immune system mediated damage, a so-called cytokine storm, and immunosuppression has been proposed for this context [95]. Furthermore, an IL6 selective monoclonal antibody is currently being tested on humans for this specific purpose [11,12]. On the basis of these elements, rather than in upfront therapy, we would consider thiopurine analogs and their associated drugs for the treatment of the cytokine storm in COVID-19, that could eventually result in an antiviral and a double immunomodulatory efficacy: inhibiting the viral replication, suppressing hyperinflammation damage and enhancing the endocellular innate immune response against SARS-cov2. We again recommend, extreme prudence in considering these drugs, as their side effects might be a danger. 

In addition, we would like to highlight that a recent report demonstrated that disulfiram (a clinically approved alcohol abuse antagonist, now repurposed as an anticancer drug) can competitively and noncompetitively inhibit SARS and MERS PLPs, respectively, and this activity is synergic with 6-thioguanine (a thiopurine) and mycophenolic acid against MERS, but not SARS, PLP [78]. These recent findings in vitro, together with a relatively good risk/benefit ratio, may make disulfiram a clinically evaluable drug against COVID-19. However, as a hepatic acetaldehyde dehydrogenase inhibitor, its pharmacological interactions might be considered.

#### 3.3.4. Proteasome and m-Calpain Inhibitors

The proteasome inhibitor MG132 has been shown to reduce cytokine levels and improve survival and lung histology in a SARS murine model [56]. The authors did not propose a strong explanation for this effect of MG132, but the reported anti-inflammatory [96] and anti-viral internalization effects [97] of the proteasome inhibitors may be the mechanisms underlying it. Interestingly, MG132 has been used in recent studies to counteract the effects induced by PLP [52,53,54,55]. This, taken together with the results in the recentmurine model of DUB-mutated PLP2 in murine hepatitis [57], suggests an involvement of PLP.

However, a mechanism has been proposed by other authors, who exclude proteasome inhibition and link MG132 antiviral activity to m-calpain (a cysteine protease) inhibition, suggesting that viral cysteine proteases, such as PLP, are possible targets of this drug. Even more promising results were obtained in vitro when the calpain specific inhibitor MDL28170 was used [98], and these data align with previous in vitro studies on calpain inhibitors in SARS [99].

## 4. Conclusions 

In summary, coronaviruses PLPs are multifunctional enzymes with cysteine protease, DUB and deISGylating activities that contribute significantly to infection pathogenesis. The efficacy of targeting PLPs as an antiviral therapy is demonstrated, at least in vitro, with further in vivo studies strongly recommended. Several compounds, both clinical and preclinical, have documented actions against these proteins, and thus may provide an attractive therapeutic strategy for COVID-19.

On the basis of these considerations, we propose four main suggestions towards the development of PLP inhibitors with the potential to treat COVID-19. The first is to consolidate our knowledge on the structure and function of SARS-CoV2 PLP in order to highlight its role in pathogenesis and facilitate drug design. Secondly, we claim that the PLPs effects may be strongly interconnected between all their enzymatic activities. Antagonizing one of these functions alone may be less effective than inhibiting the function of the whole protein. Thus, we suggest developing and testing molecules with such capability [67], rather than focusing on each of the PLPs’ functions alone [57]. However, since the PLP protease activity seems to be critical for virus replication, inhibiting at least this activity could be effective. Third, the number and the specificity of in vivo PLP inhibition models must be increased. Lastly, given the importance of a new coronavirus-specific drug in the fight against the current pandemic, the hypothesis of testing the proposed drugs in COVID-19 should not be underestimated. In detail:

- Drugs reviewed in the past [69] and novel plant derivatives should progress to the next step in pharmacological development, hopefully being evaluated in vitro against SARS-CoV2 and, subsequently, in animal models;
- Recently proposed SARS-CoV2 PLP inhibitors [3] and other drugs proposed in our review should also undergo further evaluations;
- Effects of dietary integration with plant compounds such as polyphenols on COVID-19 prevention should be investigated clinically, in order to demonstrate if this can be a relatively safe and effective health measure;
- A clinical study on the effects of high levels of cystatin C (as uremia) on the outcome of patients with COVID-19 should be performed. Trialing the exogenous administration of this peptide, i.e. via aerosol, may then be considered in humans;
- A similar strategy should be used for thiopurine analogs and disulfiram. However, the evaluation of thiopurine analogs may need to be retrospective and their administration reasoned cautiously. Disulfiram may be tested more safely, paying attention to its pharmacological interactions and any alcohol consumption.

## Figures and Tables

**Table 1:** In the table below are listed the main PLP inhibitors reported in this review.

Class	Lead Compound(s)	Target Molecule	Drug Development Stage
*Tribulus terrestris* derivates [71]	Terrestrimine	SARS-CoV PLP	Preclinical
*Angelica keiskei* derivates [72]	Xanthoangelol E	SARS-CoV 3CLP and PLP-whole enzymatic activity	Preclinical
*Broussonetia Papyrifera* derivates [73]	Papyriflavonol A	SARS and MERS-CoV 3CL and whole PLP	Preclinical
*Psoralea Corylifolia* derivates [74]	Isobavachalcone	SARS-CoV PLP	Preclinical
	Psoralidin		
*Strobilanthes cusia* derivates [75]	Tryptanthrin	hCoV-NL63 (PLP2-proposed)	Preclinical
	Indigodole B		
Thiopurine analogs [76,77]	6-Thioguanine	SARS-CoV PLP protease and MERS-CoV PLP protease and DUB	Clinically used
	6-Mercaptopurine		
Acethaldeyde dehydrogenase inhibitor [78]	Disulfiram	SARS and MERS-CoV PLPs	Clinically used
Endogenous peptide [79]	Cystatin C	Human coronaviruses OC43 and 229e (PLPs proposed)	Endogenous Peptide
